# ANCA negative eosinophilic granulomatosis with polyangiitis (EGPA) presenting with left ventricular thrombus: An appreciation of distinct phenotypes and eosinophilic driven pathogenesis

**DOI:** 10.1002/rcr2.1033

**Published:** 2022-09-02

**Authors:** Kai Chaivannacoopt, Eliza Flanagan

**Affiliations:** ^1^ Respiratory Department University Hospital Geelong Geelong Australia

**Keywords:** ANCA negative, EGPA, eosinophilic granulomatosis with polyangiitis, left ventricular thrombus

## Abstract

Eosinophilic granulomatosis with polyangiitis (EGPA) is a rare multisystem disorder, included in the spectrum of the antineutrophil cytoplasmic antibodies (ANCA) associated vasculitides. There are heterogeneous clinical features and a lack of consensus in standardized diagnostic criteria, with an underappreciation of eosinophilic manifestations. There are now reported phenotypical differences between ANCA‐positive and negative EGPA, with myocardial involvement, lung infiltrates and gastrointestinal symptoms predominating in ANCA‐negative cases. We report a rare presentation of ANCA‐negative EGPA in a woman with respiratory, neurological and cardiac involvement, manifesting as a large left ventricular thrombus without significant cardiac dysfunction.

## Graphical abstract

Eosinophilic granulomatosis with polyangiitis (EGPA) is a rare multisystem disorder, included in the spectrum of the antineutrophil cytoplasmic antibodies (ANCA) associated vasculitides. There are now reported phenotypical differences between ANCA‐positive and negative EGPA. We report a rare presentation of ANCA‐negative EGPA in a woman manifesting as a large left ventricular thrombus.

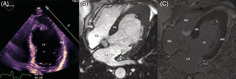

## INTRODUCTION

Eosinophilic granulomatosis with polyangiitis (EGPA) is a rare multisystem disorder historically characterized by asthma, rhinosinusitis and peripheral blood eosinophilia.^1^, ^2^

Included in the spectrum of the antineutrophil cytoplasmic antibodies (ANCA) associated vasculitides, EGPA is a disease that borders between primary systemic vasculitides and hypereosinophilic disorders.^3^ There continues to be heterogeneous clinical features and a lack of consensus in standardized diagnostic criteria.

Cardiac involvement occurs in 15%–60% of cases and is an important predictor of mortality. Left ventricular thrombus appears to be an underappreciated manifestation in EGPA.^4^

## CASE REPORT

A 62‐year‐old woman with a background of hypertension and eosinophilic asthma was admitted with severe exertional dyspnoea and productive cough. She reported deterioration in her asthma control over the preceding months, despite systemic oral corticosteroids. This was in the setting of having asthma for over 30 years with stable symptoms on fluticasone (250 mcg) and salmeterol (25 mcg) combination therapy.

There was longstanding sinus congestion, thought to be allergic in nature, without nasal polyps, epistaxis or haemoptysis. Approximately 6 months prior, she had developed right second toe numbness and more recently tinnitus without hearing loss. She had no significant rashes, livedo reticularis, arthralgias, myalgias, scleritis, lower limb weakness or back pain. There was no gastrointestinal, cardiac or constitutional symptoms and no history of thrombosis or miscarriage.

Physical examination demonstrated a mild bilateral wheeze and reduction in light touch sensation to her right second toe. All vital signs were within normal limits.

Urine was bland, with no proteinuria or sediment. Creatinine was 69 μmol/L. White cell count was 11.1 × 10^9^ cells L^−1^ with an eosinophilia of 1.8 × 10^9^ cells L^−1^. Haemoglobin was 138 g/L and C‐reactive protein was normal at 3 mg/L. IgE was elevated at 286 KU/L. Anti‐nuclear antibody was borderline at 1:160. Extractable nuclear antigens and troponin were negative. p‐ANCA was indeterminant with no detectable PR3 or MPO titres. Electrocardiogram showed a left bundle branch block.

She was commenced on hydrocortisone, nebulised bronchodilators and broad‐spectrum antibiotics with good response.

**FIGURE 1:**
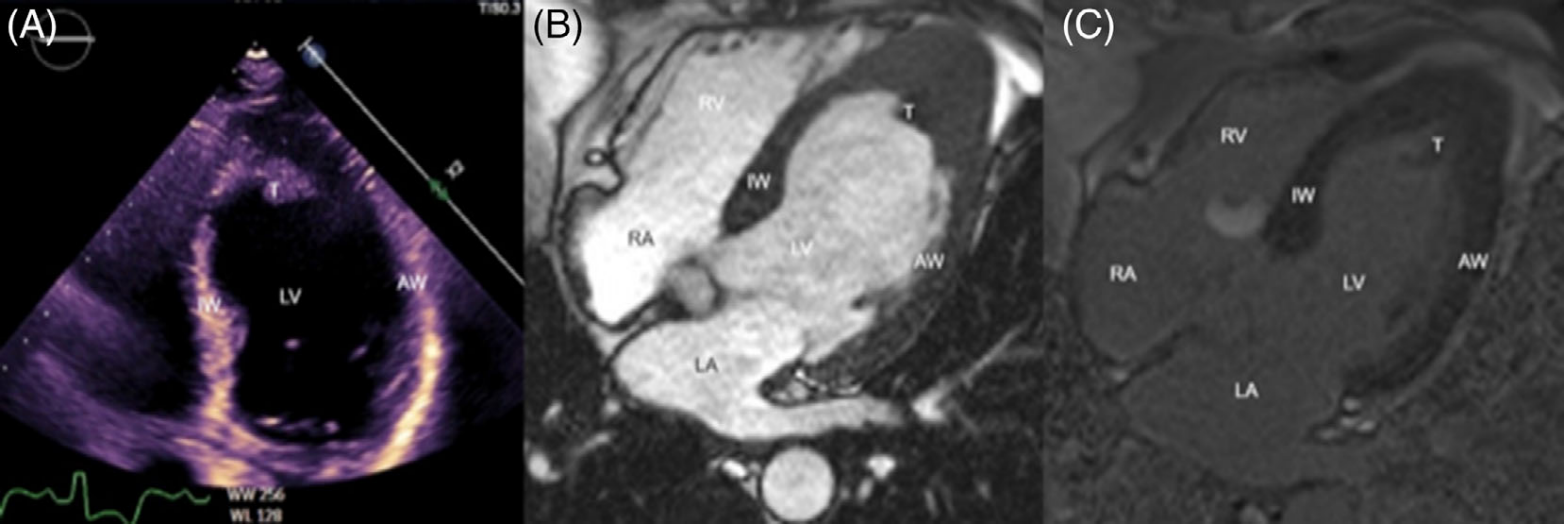
Apical 4 chamber view on transthoracic echocardiogram (A) and cardiac magnetic resonance (cMRI) imaging pre gadolinium cine image (B) and post gadolinium (C). IW, inferoseptal wall; AW, anterolateral wall; T, thrombus; LV, left ventricle; LA, left atrium

**FIGURE 2:**
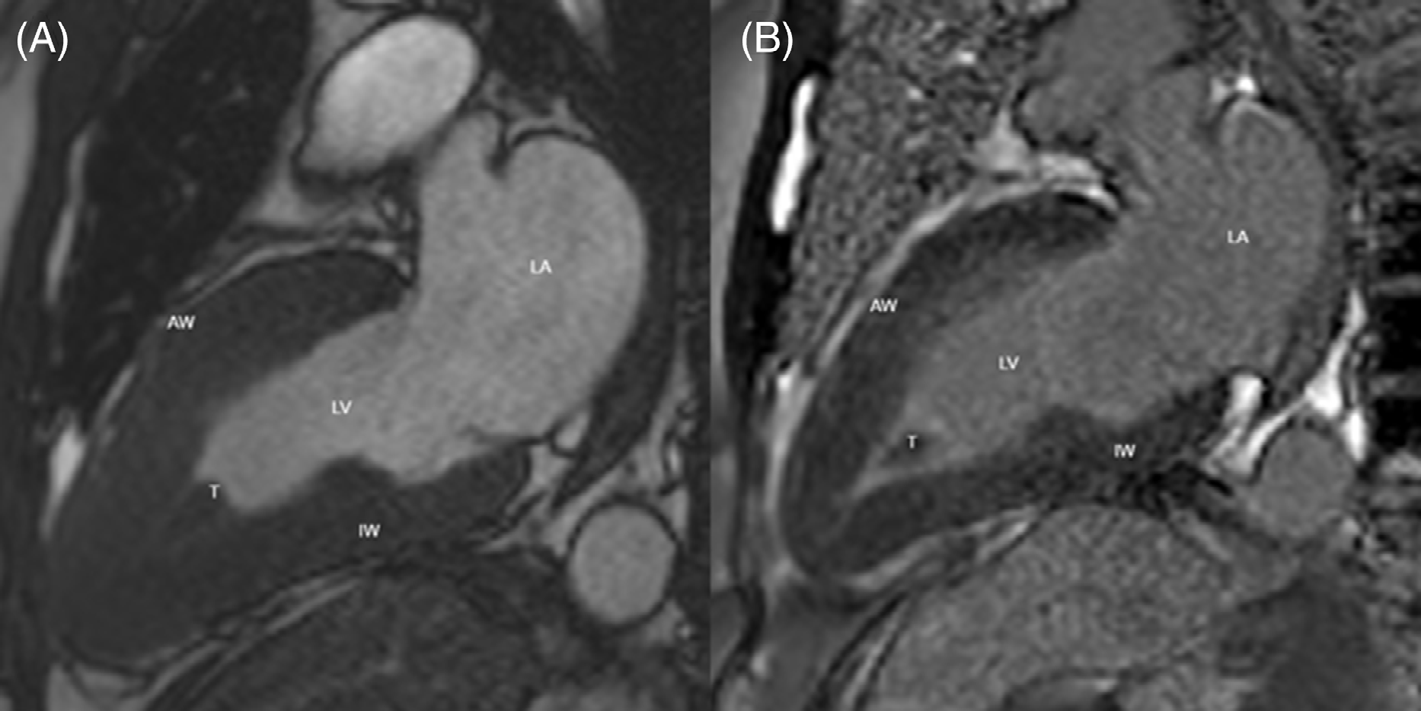
Long axis 2 chamber view on cardiac magnetic resonance imaging (cMRI) pre gadolinium cine image (a) and post gadolinium (b). IW, inferoseptal wall; AW, anterolateral wall; T, thrombus; LV, left ventricle; LA, left atrium; RV, right ventricle; RA, right atrium

CT chest showed peripheral micro‐nodularity and subpleural reticulations. CT of her sinuses showed minimal mucosal thickening. Transthoracic echocardiogram (TTE) displayed preserved left ventricular systolic function and a large left ventricular thrombus (Figure 1). Cardiac MRI demonstrated a large left ventricular thrombus occupying much of the apical lumen, with subtle subendocardial enhancement on the inferior and septal wall of the left ventricular apex (Figures 1 and 2). Overall findings were thought to be in keeping with eosinophilic myocarditis.

MRI brain and abdominal ultrasound were unremarkable. FIP1L1‐PDGFRA was negative to exclude clonal hypereosinophilia.

Diagnosis of EGPA was made with worsening asthma symptoms, presence of eosinophilia, peripheral neuropathy and eosinophilic myocarditis with resultant left ventricular thrombus formation. This would satisfy the required 4 of 6 American College of Rheumatology (ACR) classification criteria with a prognostic Five‐Factor Score of 0.^1^

Systemic anticoagulation with warfarin, intravenous pulse methylprednisolone 1 g daily and an initial dose of 1.5 g intravenous cyclophosphamide were commenced with improvement in symptoms.

Cyclophosphamide infusions were continued monthly, with planned reassessment after her sixth dose, along with slow wean of oral corticosteroids. Repeat TTE at 3 months showed reduction in size of left ventricular thrombus.

## DISCUSSION

EGPA remains a significant differential in severe asthma diagnostic algorithms.

Multiple sets of criteria for diagnosis and classification of EGPA have been proposed with asthma, eosinophilia and evidence of vasculitis forming the foundations of these criteria.^2^ These criteria often require biopsy‐proven vasculitis, which is not always possible. ANCA itself can be used as surrogate marker of small vessel vasculitis, but the absence of ANCA does not exclude the diagnosis.^2^

ANCA are only present in 40% of EGPA patients and are primarily myeloperoxidase (MPO) positive p‐ANCA.^1^ EGPA is a disease that borders the primary systemic vasculitides and hypereosinophilic disorders. There are now reported phenotypical differences between ANCA‐positive and negative EGPA; the former presenting with classical glomerulonephritis, alveolar haemorrhage, mononeuritis multiplex and ear, nose and throat disease. Myocardial involvement, lung infiltrates and gastrointestinal symptoms predominate in ANCA‐negative cases.^1^

Hypereosinophilic syndrome (HES) remains the significant differential diagnosis. HES has well‐documented manifestations including thrombosis.^3^ Discrimination is difficult without histology. In our case, the eosinophilia remained modest, and just meets criteria for hypereosinophilia on peripheral blood samples to satisfy ACR criteria.^3^, ^5^ The degree of hypereosinophilia may have been blunted by systemic corticosteroid use, predating hospital presentation. Eosinophil activation is known to causes tissue fibrosis, thrombosis or both, and is thought to be the primary driver in EGPA related cardiac injury over the ANCA mediated inflammation.^3^

Cardiac involvement in EGPA occurs in 15%–60% of cases and has a wide variety of manifestations.^4^ These are predominantly pericarditis and cardiomyopathy, the latter of which confers a poorer prognosis.^6^ Coronary vasculitis and intraventricular thrombosis are particularly rare.^4^ Intraventricular thrombus has been documented in both ANCA positive and negative case studies.^7^, ^8^, ^9^ Cardiac involvement has also been linked to insufficient non‐corticosteroid immunosuppression.^7^ Emergence of cardiac MRI may identify an underappreciated incidence of cardiac thrombosis and fibrosis indicative of EGPA, not seen on transthoracic echocardiogram. Late gadolinium enhancement (LGE) has allowed demonstration of non‐ischaemic lesions including focal fibrosis.^10^

The Five‐Factor score for prognosis in systemic vasculitides includes cardiac involvement as a scoring parameter. This scoring system does not include pathology found on investigations in the absence of symptoms, as in our case.^6^ Untreated left ventricular thrombus however, poses a substantial risk of potential thromboembolic complications.

EGPA has also been documented to present with three phases; beginning with asthma, followed by tissue eosinophilia and finally small‐vessel vasculitis.^10^ The relationship of these phases to patient ANCA status is unclear. Given this natural history of EGPA progression, there is some suggestion that early immunosuppression may be beneficial in preventing both eosinophilic and vasculitic sequalae.

This case highlights cardiac thrombus as a rare manifestation for EGPA, particularly ANCA negative disease where eosinophilic infiltration appears to predominantly drive disease processes. It enforces the ongoing need to consider EGPA as a differential diagnosis in severe allergic asthma and highlights the potentially severe eosinophilic manifestations of the disease, in addition to conventional vasculitic phenomenon.

Finally, it reflects a need for more accurate and practical diagnostic and classification criteria.

## AUTHOR CONTRIBUTION

Conception for submission as a case report was established by the Kai Chaivannacoopt. Communication with the patient, collection of relevant information, correspondence and investigations as well as literature review was also conducted by the Kai Chaivannacoopt. Initial draft and critical revision was performed by both the Kai Chaivannacoopt and Eliza Flanagan, with agreement for submission of the final article.

## CONFLICT OF INTEREST

None declared.

## ETHICS STATEMENT

The authors declare that appropriate written informed consent was obtained for the publication of this manuscript and accompanying images.

## Data Availability

Data sharing is not applicable to this article as no new data were created or analyzed in this study.

## References

[1] Greco A , Rizzo MI , De Virgilio A , Gallo A , Fusconi M , Ruoppolo G , et al. Churg‐Strauss Syndrome. Autoimmun Rev. 2015;14:341–8. 25500434 10.1016/j.autrev.2014.12.004

[2] Mouthon L , Dunogue B , Guillevin L . Diagnosis and classification of eosinophilic granulomatosis with polyangiitis (formerly Churg‐Strauss syndrome). J Autoimmun. 2014;48‐49:99–103. 10.1016/j.jaut.2014.01.018 24530234

[3] Valent P , Kilon AD , Horny HP , Roufosse F , Gotlib J , Weller PF , et al. Contemporary consensus proposal on criteria and classification of eosinophilic disorders and related syndromes. J Allergy Clin Immunol. 2012;130:607–12. 22460074 10.1016/j.jaci.2012.02.019 PMC4091810

[4] Miloslavsky E , Unizony S . The heart in vasculitis. Rheum Dis Clin N Am. 2014;40:11–26. 10.1016/j.rdc.2013.10.006 24268007

[5] Masi AT , Hunder GG , Lte JT , Michel BA , Bloch DA . The American College of Rheumatology 1990 criteria for classification of Churg‐Strauss syndrome (allergic granulomatosis and angiitis). Arthritis Rheumatol. 1990;8(33):1094–100. 10.1002/art.1780330806 2202307

[6] Guillevin L , Pagnoux C , Seror R , Mahr A , Le Toumelin P . The five‐factor score revisited; assessment of prognoses of systemic necrotizing vasculitides based on the French vasculitis study group (FVSG) cohort. Medicine. 2011;90(1):19–27. 21200183 10.1097/MD.0b013e318205a4c6

[7] Zhu D , Luo Y , Liu X , Zu L . Antiproteinase 3 positive eosinophilic granulomatosis with polyangiitis with heart failure and intraventricular thrombosis. Case Rep Rheumatol. 2017;2017:1–3. 10.1155/2017/2908185 PMC5303599 28251013

[8] Saito Y , Okada S , Funabashi N , Kobayashi Y . ANCA‐negative eosinophilic granulomatosis with polyangiitis (EGPA) manifesting as a large intracardiac thrombus and glomerulonephritis with angionecrosis. BMJ Case Rep. 2016;2016:bcr2016216520. 10.1136/bcr-2016-216520 PMC5020759 27591039

[9] Hamudi J , Karkabi B , Zisman D , Shiran A . Severe biventricular thrombosis in eosinophilic granulomatosis with polyangiitis: a case report. Eur Heart J Case Rep. 2020;4:1–5. 10.1093/ehjcr/ytaa417 PMC7793161 33442628

[10] Cereda AF , Pedrotti P , De Capitani L , Giannattasio C , Roghi A . Comprehensive evaluation of cardiac involvement in eosinophilic granulomatosis with polyangiitis (EGPA) with cardiac magnetic resonance. Eur J Intern Med. 2017;39:51–6. 27727077 10.1016/j.ejim.2016.09.014

